# Recent Advances in Systems and Network Medicine: Meeting Report from the First International Conference in Systems and Network Medicine

**DOI:** 10.1089/sysm.2020.0001

**Published:** 2020-02-26

**Authors:** Emma Kurnat-Thoma, Ancha Baranova, Pat Baird, Elia Brodsky, Atul J. Butte, Amrita K. Cheema, Feixiong Cheng, Shuchismita Dutta, Christina Grant, James Giordano, Anke H. Maitland-van der Zee, Douglas B. Fridsma, Robert Jarrin, Maricel G. Kann, Jonathon Keeney, Joseph Loscalzo, Guru Madhavan, Bradley A. Maron, Dennis K. McBride, Maeve McKean, Seong K. Mun, James C. Palmer, Bakul Patel, Kapil Parakh, Anne R. Pariser, Christian Pristipino, Timothy R.D.J. Radstake, Harsha K. Rajasimha, William B. Rouse, Damjana Rozman, Alif Saleh, Harald H.H.W. Schmidt, Nikolaus Schultz, Tavpritesh Sethi, Edwin K. Silverman, Jessica Skopac, Igor Svab, Sylvia Trujillo, James E. Valentine, Dinesh Verma, Bruce J. West, Sona Vasudevan

**Affiliations:** ^1^ National Institutes of Health, National Institute of Nursing Research (NIH/NINR), Bethesda, Maryland.; ^2^ Genetics and Genomics, Georgetown University School of Nursing and Health Studies, Washington, District of Columbia.; ^3^ The Chronic Metabolic and Rare Diseases Systems Biology Initiative (ChroMe RaDSBIn), School of Systems Biology, George Mason University, Fairfax, Virginia.; ^4^ Global Software Standards, Philips, Pleasant Prairie, Wisconsin.; ^5^ Pine Biotech, New Orleans, Louisiana.; ^6^ Bakar Computational Health Sciences Institute, University of California San Francisco (UCSF), San Fransisco, California.; ^7^ Department of Biochemistry, Molecular and Cellular Biology, Georgetown University Medical Center, Washington, District of Columbia.; ^8^ Cleveland Clinic Lerner College of Medicine, Case Western Reserve University, Cleveland Clinic, Cleveland, Ohio.; ^9^ Research Collaboratory for Structural Bioinformatics Protein Data Bank (RCSB PDB) and Institute for Quantitative Biomedicine, Rutgers University, The State University of New Jersey, Piscataway, New Jersey.; ^10^ Rare Disease Institute, Division of Genetics and Metabolism, Children's National Medical Center, Washington, District of Columbia.; ^11^ Department of Neurology, Neuroethics Studies Program-Pellegrino Center for Clinical Bioethics, and O'Neill-Pellegrino Program in Brain Sciences and Global Law and Policy, Georgetown University Medical Center, Washington, District of Columbia.; ^12^ Department of Biochemistry, Neuroethics Studies Program-Pellegrino Center for Clinical Bioethics, and O'Neill-Pellegrino Program in Brain Sciences and Global Law and Policy, Georgetown University Medical Center, Washington, District of Columbia.; ^13^ Department of Respiratory Medicine, Amsterdam University Medical Centers, University of Amsterdam, Amsterdam, The Netherlands.; ^14^ American Medical Informatics Association, Washington, District of Columbia.; ^15^ Department of Biological Sciences, University of Maryland Baltimore County, Baltimore, Maryland.; ^16^ Department of Biochemistry and Molecular Medicine, George Washington University, Washington, District of Columbia.; ^17^ Department of Medicine, Brigham and Women's Hospital and Harvard Medical School, Boston, Massachusetts.; ^18^ National Academy of Engineering, The National Academies of Sciences, Engineering, and Medicine, Washington, District of Columbia.; ^19^ Center for Advanced Healthcare Learning and Simulation (CAHLS), Washington, District of Columbia.; ^20^ Georgetown University Global Health Initiative, Georgetown University Medical Center, Washington, District of Columbia.; ^21^ Arlington Innovation Center, Health Research, Virginia Tech, Arlington, Virginia.; ^22^ Caldwell Palmer, Denver, Colorado.; ^23^ Center for Devices and Radiological Health, Food and Drug Administration, White Oak, Maryland.; ^24^ Yale School of Medicine, New Haven, Connecticut.; ^25^ National Institutes of Health, National Center for Advancing Translational Sciences (NIH/NCATS), Bethesda, Maryland.; ^26^ Interventional Cardiology Unit, San Filippo Neri ASL Roma 1 Hospital, Rome, Italy.; ^27^ Italian Association for Systems Medicine and Healthcare (ASSIMSS), Rome, Italy.; ^28^ Department of Rheumatology, Clinical Immunology and Center of Translational Immunology, University Medical Center Utrecht, Utrecht, The Netherlands.; ^29^ Jeeva Informatics Solutions, Inc., Reston, Virginia.; ^30^ McCourt School of Public Policy, Georgetown University, Washington, District of Columbia.; ^31^ Faculty of Medicine, University of Ljubljana, Ljubljana, Slovenia.; ^32^ Scipher Medicine, Boston, Massachusetts.; ^33^ Department of Pharmacology & Personalized Medicine, Faculty of Health, Medicine & Life Sciences, Maastricht University, Maastricht, The Netherlands.; ^34^ Department of Epidemiology and Biostatistics, Memorial Sloan Kettering Cancer Center, New York, New York.; ^35^ Department of Computational Biology, Indraprastha Institute of Information Technology Delhi (IIIT), Delhi, India.; ^36^ MITRE Corporation, McLean, Virginia.; ^37^ Life Sciences/Digital Medicine, American Medical Association, Washington, District of Columbia.; ^38^ Hyman, Phelps & McNamara PC, Washington, District of Columbia.; ^39^ University of Maryland Carey School of Law, Baltimore Maryland.; ^40^ Systems Engineering Research Center (SERC), Stevens Institute of Technology, New Jersey.; ^41^ Army Research Laboratory, Army Research Office, Durham, North Carolina.

**Keywords:** network medicine, international conference, systems, big data, artificial intelligence, ethical legal social implications (ELSI), regulatory and health policy

## Abstract

The *First International Conference in Systems and Network Medicine* gathered together 200 global thought leaders, scientists, clinicians, academicians, industry and government experts, medical and graduate students, postdoctoral scholars and policymakers. Held at Georgetown University Conference Center in Washington D.C. on September 11–13, 2019, the event featured a day of pre-conference lectures and hands-on bioinformatic computational workshops followed by two days of deep and diverse scientific talks, panel discussions with eminent thought leaders, and scientific poster presentations. Topics ranged from: Systems and Network Medicine in Clinical Practice; the role of -omics technologies in Health Care; the role of Education and Ethics in Clinical Practice, Systems Thinking, and Rare Diseases; and the role of Artificial Intelligence in Medicine. The conference served as a unique nexus for interdisciplinary discovery and dialogue and fostered formation of new insights and possibilities for health care systems advances.

## Introduction

The *First International Conference in Systems and Network Medicine* was held at Georgetown University's Conference Center from September 11 to 13, 2019. We had an exciting agenda that included topics on the application of Systems and Network Medicine in Clinical Practice; the role of -omics technologies in Health Care; the role of Education and Ethics in Clinical Practice, Systems Thinking, and Rare Diseases; and the role of Artificial Intelligence in Medicine. Conference sessions were deep and diverse. In addition to the talks, we had two panel discussions covering important topics on the challenges in the health care systems and role of digital medicine, with eminent leaders in the field who served as panelists.

The 3-day exciting event was hosted by Georgetown University's Systems Medicine Program and MedStar Institute of Innovation and co-hosted by the International Association of Systems Medicine and the European Association for Systems Medicine. This event would not have been possible without the help of the generous sponsors. The goal of this article is to summarize the key highlights from the conference that brought together close to 200 attendees that included global thought leaders, scientists, clinicians, academicians, industry and government experts, medical and graduate students, postdoctoral scholars, and policymakers.

## Day 1: Preconference Workshop

The conference began with a preconference workshop featuring several hands-on computational sessions led by experts in the field. The day-long learning ended with a networking event that, among other things, exposed students to potential career paths.

The preconference workshop featured two lectures outlining the state of the science in precision theranostics, which is a new field in medicine that uses specific biological pathways in the human system and chronic obstructive pulmonary disease (COPD) by eminent experts in the field of systems medicine, Harald H.H.W. Schmidt, MD, PhD, PharmD, and Edwin K. Silverman, MD, PhD. Talks were followed by hands-on computational science practice sessions for participants led by health care systems artificial intelligence (AI) technology, regulatory, -omics integration, and proteomic precision medicine content experts Tavpritesh Sethi, MBBS, PhD, IIIT, Jonathan Keeney, PhD, Elia Brodsky, CEO, and Shuchismita Dutta, PhD.

### Mechanism-based disease definitions for precision theranostics

*Harald H.H.W. Schmidt, MD, PhD, PharmD,* is the chairman of pharmacology and personalized medicine at the School for Mental Health and Neuroscience, Maastricht University, The Netherlands. Dr. Schmidt presented his perspectives on the end of medicine as we know it stemming from his pioneering work in disease redefinition, drug repurposing, and big data for network pharmacology. He summarized how the current practice of medicine depends on late diagnosis of disease by the detection of symptoms at an organ level, but in most cases not by a mechanistic understanding of disease. This results in imprecise and high number to treat approaches.

Network medicine aims to define diseases by causal molecular mechanism allowing for precise diagnosis and precise intervention with low numbers needed to treat. These mechanisms are not single targets but signaling modules within the interactome. To fully cover disease causality, however, our exposome and microbiome need to be taken into account to prevent unnecessary drug interventions where, for example, lifestyle changes would be the most effective approach. Network medicine will thus overcome the limitations of symptom-based medicine that treats disease to precision medicine that cures disease.

### From genetics to network medicine in COPD

*Edwin Silverman, MD, PhD*, is a professor of medicine at Harvard Medical School and chief of the Channing Division of Network Medicine at Brigham and Women's Hospital. He has participated in collaborative genome-wide association studies (GWASs) that have identified >80 genomic regions associated with COPD; however, the identification of functional genetic variants within associated loci remains challenging. Gene-targeted murine models, integration of -omics data, and functional variant analyses of these GWAS regions have provided some important insights into COPD pathogenesis. However, single genetic variants are unlikely to explain complex diseases such as COPD, because perturbations of biological networks and not isolated genes confer complex disease risk. In gene expression analysis of lung tissue samples, the top COPD GWAS loci were not differentially expressed. However, genes that interact with COPD GWAS genes, including HHIP, FAM13A, and IREB2, were often differentially expressed in lung tissue.

Using COPD GWAS genes as seed genes for random walk analysis within the protein–protein interaction network, a COPD disease network module (composed of 163 genes) was created that had significant differences in gene expression between COPD cases and controls in multiple disease-relevant samples. Correlation-based networks, gene regulatory networks, and protein–protein interaction networks can provide complementary information regarding complex diseases.

### From complex networks to clinical decisions with Bayesian AI

*Tavpritesh Sethi, MBBS, PhD,* is a physician-scientist and assistant professor of computational biology at Indraprastha Institute of Information Technology, Delhi, India; a fellow of the Wellcome Trust/DBT India Alliance. He outlined how networks are the most intuitive representations of data. However, they rely upon pairwise decisions that limit their utility. Bayesian Decision Networks (BDNs) extend a class of probabilistic graphical models (Bayesian Networks) through decision theory and are used regularly in business settings. However, BDNs are underused in clinical and public health settings due to data set complexity. Dr. Sethi guided workshop participants through a hands-on Bayesian-learning module to provide practice with linked-open data from clinical sepsis and public health settings. Bootstrap evaluations, quantitative inferencing, and optimal decision frameworks for model deployment using a web-based application (R/Shiny) gave participants an introduction to Explainable Artificial Intelligence and Fair Accountable Transparent Machine Learning using the open-source platform *wiseR*.

### PrecisionFDA challenge: Creating and submitting BioCompute objects

*Jonathon Keeney, PhD*, is the managing director of the Executive Steering Committee of the BioCompute Public Private Partnership and an assistant research professor at George Washington University. BioCompute is a mechanism to record and communicate entire bioinformatic workflows. BioCompute partitions information in the workflow into conceptually meaningful categories, including both space for high-level overview in the form of free text, and granular details, such as software version numbers and parameters. BioCompute takes guesswork out of communication, outlines a structure for a more predictable flow of information, and substantially improves the ability to communicate bioinformatics analysis pipelines, thus allowing them to be clearly understood and easily reproduced by others. An instance of a bioinformatic workflow (scripts, codes, processes, gene variant annotations, etc.), that is created in a way that adheres to the BioCompute specification, is called a “BioCompute Object” or BCO.

PrecisionFDA is a platform hosted by the U.S. Food and Drug Administration (FDA) that allows users to develop their own bioinformatic workflows and submit BCOs and associated tools for FDA review. Dr. Keeney walked participants through the process of choosing an existing workflow from literature, and capturing and creating a BCO from it using a free online tool called the BCO Editor. In addition to Dr. Keeney, Dr. Raja Mazumder, PhD, from George Washington University, Mr. Hadley King from George Washington University, and Ms. Holly Stephens from PrecisionFDA contributed to the presentation.

### From data to interpretation-mining data for answers about health and disease

*Elia Brodsky, MBA,* is the cofounder and CEO of Pine Biotech, a company that merges big -omics data analysis with clinical care applications and real-world evidence. Multiple integration is providing valuable insights into molecular subtypes of various diseases. The methodologies behind integration, feature selection, and interpretation of -omics data is evolving rapidly.

Mr. Brodsky guided participants through several examples of multiomics data integration, including genomic, transcriptomic, proteomic, metabolomic, and microbiome information, for several selected diseases. Specific examples included gene expression patterns in breast cancer and miRNAs in hepatocellular carcinoma (HCC). A number of specific analytic methods and machine learning techniques for -omics data integration were reviewed, including principal component analysis, factor regression analysis, decision trees, random forests, bagging and bootstrapping, two-way orthogonal partial least squares analysis, and partial least squares analysis. Key aspects of data processing were summarized and known factors impacting reproducibility and validation were discussed.

### Molecular medicine with three-dimensional insights

*Shuchismita Dutta, PhD,* is the scientific educational development lead at Research Collaboratory for Structural Bioinformatics Protein Data Bank (RCSB PDB), and associate research professor at the Institute for Quantitative Biomedicine at Rutgers University, NJ. She introduced participants to the biomolecular structural data, tools, and resources available from RCSB PDB.

Vast amounts of heterogeneous data spanning genomics, proteomics, metabolomics, transcriptomics, and digital health information enables personalized assessments and provide management options for the practice of precision medicine. Visualization of three-dimensional (3D) shapes and interactions of key molecules involved in disease processes can help facilitate multidisciplinary collaboration to present novel insights about diseases and guide the design of new therapeutic rationales. The PDB provides access to experimentally determined 3D structures for >155,000 biological macromolecules and their various complexes. Workshop participants were introduced to freely available PDB tools and resources to visualize, explore, and analyze molecular structures.

Participants gained experience with the identification of macromolecules and small molecules contributing to the development of a number of diseases, including sickle cell disease, type 2 diabetes, and lung adenocarcinoma.

Day 1 concluded with a networking event organized by Dr. Caleb McKinney, assistant dean of graduate and postdoctoral training and development, Georgetown University Medical Center. This event was attended by medical and graduate students and postdoctoral scholars. We would like to acknowledge the contributions of the special guests to this event: Ms. Alyssa Parks, Lupus Foundation of America, Dr. Dawn Beraud, National Institutes of Health, Ms. Jaclyn Levy, Infectious Society of America and, Ms. Taylor Schulte, Georgetown University to the event.

## Day 2: Plenary Talks, Keynote Addresses, Panel Discussions, and Session Talks

The day began with morning prayers offered by Fr. Jerry Hayes, SJ, director of Ignatian programs, Office of Mission and Ministry, Georgetown University. This was followed with a welcome message by conference chair, Dr. Sona Vasudevan, director of Georgetown University Medical Center's Systems Medicine Program and professor, department of biochemistry and molecular cellular biology. Dr. Vasudevan extended a warm welcome to all the delegates on behalf of the organizing committee, both local and international, for valued contributions and participation to make the conference a great success! Dr. Vasudevan's message was followed by Dr. Elliott Crooke, senior associate dean of faculty and academic affairs, Georgetown University's Medical Center, who thanked the attendees for coming.

### Plenary session 1, address 1: “Health informatics: systems medicine for the 21st century”

*Doug Fridsma, MD, PhD, FACP, FACMI,* the president and CEO of American Medical Informatics Association, opened the session with a plenary talk providing a 360° view of current health care challenges, role of informatics, and the need for systems-level integration. Dr. Fridsma put the patient experience at the center while speaking about the role of informatics for clinicians, businesses, and health care organizations. He presented compelling cases showing that health informatics has matured to the point that a new breed of health IT professionals needs to emerge.

These professionals will not only need to be adept at data management, but will also need to understand the science of extracting information from data that will empower patients. Creating value for patients will also empower entrepreneurs to create products and services that patients will use to make decisions about their own health. Finally, Dr. Fridsma alluded to challenges in a rapidly changing world where data ownership and rights will become increasingly important. The plenary session ended on a highly positive note with the audience enthralled with the opportunities that lie ahead in applying Systems Medicine approaches to deliver better health care.

### Plenary session 1, address 2: “Network medicine: approach to the definition, diagnosis, and treatment of disease in the era of precision medicine”

*Joseph Loscalzo, MD, PhD,* gave the second plenary talk. Dr. Loscalzo is a professor at Brigham and Women's Hospital, Harvard Medical School, and a pioneer of Network Medicine.

Dr. Loscalzo began his plenary lecture by placing recent biomedical advances in a historical context. Since the 19th century, clinicians and biomedical researchers have used the Cartesian reductionist approach to study human biology, disease, and therapeutics. He stated that although the success of this strategy is indisputable, it has major shortcomings, including most importantly oversimplification of complex biological phenomena. Until recently, the prospect of unraveling that complexity in a more integrative way has been limited by restricted data sets and inadequate analytical approaches. Dr. Loscalzo identified that biomedicine is now, however, poised to explore rigorously integrative system responses that govern *pathophenotype*, or pathology associated with a particular disease. He outlined how rapid growth in large genomic data sets and detailed phenotyping coupled with the rapid expansion of quantitative approaches to their network-based analysis provide a unique opportunity by which to define the response of biological systems to normal, pathological, and therapeutic perturbations.

Biomedical science is, therefore, now positioned to explore pathobiological complexity directly. He provided evidence on how the new field of *network medicine*, which applies systems biology and network science approaches to the dissection of molecular pathobiology and treatment, offers a truly novel path toward (re)defining and treating human disease in the modern era, and facilitates the trajectory of true precision medicine.

### Session 1: Systems/network medicine in clinical practice

#### Systems medicine in immune-mediated disease: Opportunities for drug discovery and molecular classification of disease (keynote address)

*Timothy R.D.J. Radstake, MD, PhD,* from University Medical Center, Utrecht, The Netherlands, presented his group's highly impactful work of setting the stage for use of large cohort studies and realization of the breadth of -omics integration and clinical utility. Dr. Radstake presented pathbreaking research from his group in autoimmune diseases such as scleroderma. The talk focused on learning complex immune networks from large patient cohorts in 15 different diseases and their validation through meta-analyses. The key underpinning of his talk was the integration of information across multiomic layers to arrive at clinically predictive and explainable mechanisms learned from >2000 patients. These mechanisms are largely shared across many diseases and could relate back to the *intermediate pathophenotypes* alluded to earlier in the day.

Dr. Radstake's talk also highlighted the need for reclassifying diseases through molecular networks that permeate different immune cells, including T cells, B cells, monocytes, and myeloid and plasmacytoid dendritic cells. The common emphasis on shared etiological features rather than shared phenotypic presentations in two different specialties, that is, cardiology and rheumatology, underscored the integrative nature of Systems Medicine that cuts across disciplines.

#### Precision medicine in respiratory disease: Are we beyond fiction?

*Anke-Hilse Maitland-Van Der Zee, PhD,* from Amsterdam University Medical Centers, The Netherlands, discussed upcoming computational approaches such as Similarity Network Fusion (SNF) analysis, which allows for the integration of diverse sets of information from various -omics and clinical layers to stratify asthma patients. The impactful work on disentangling asthma subphenotypes further revealed that it was possible to achieve higher statistical significance despite small sample sizes by using SNF instead of reductionist approaches. She also showed the rich information content within convenient to obtain matrices such as exhaled breath, thus democratizing the use of systems approaches for clinics without the need for complex biopsies or invasive tests.

#### Network medicine, risk stratification, and pulmonary hypertension

*Bradley A. Maron, MD,* from Brigham and Women's Hospital, Harvard Medical School, then spoke about unexplained dyspnea as a harbinger of pulmonary hypertension and the opportunity for early detection offered by the networks approach that he developed. In this clinical study, Dr. Maron collected and used invasive cardiopulmonary testing data to construct a correlation network.^1^ From the exercise network, a subnetwork was developed that included peak volume of oxygen consumption and nine other variables.

Information from the subnetwork yielded clusters that were predictive of subsequent clinical events, and in this regard outperformed standard prediction analysis methods such as logistic regression. This networks-based feature selection led to clinical utility in the form of a point-of-care risk stratification calculator for patients with exercise intolerance. Most importantly, the findings of networks-driven risk score were validated in two cohorts across different continents (North America and Europe) for predicting hospitalization in patients with unexplained exercise intolerance.

#### Applying network medicine to change the way patients with autoimmune diseases are treated

*Alif Saleh, MS,* continued the theme of network-based subphenotype discovery. Mr. Alif Saleh's talk emphasized early detection of nonresponders to first-line antitumor necrosis factor (ANF) therapy for autoimmune disease treatment. Mr. Saleh, who is the CEO of Scipher Medicine Corporation, described the health and economic costs associated with failure of ANF therapies. He further demonstrated how Scipher Medicine's network-based approaches identify response gene expression signatures and predict nonresponders with high accuracy. Being able to identify nonresponders and putting them on right alternative therapy from day 1, improves patient outcome and saves significant amount of dollars currently wasted in ineffective therapy.

#### Systems research, clinical practice and management: The Italian road to systems medicine

*Christian Pristipino, MD,* gave the final talk of this session. It was a tour de force of the Italian Road to Systems Medicine as choreographed by Dr. Christian Pristipino, founding president of Italian Association of Systems Medicine (ASSIMSS). He unveiled the extraordinary convergence of 26 experts from medicine, nursing, systems science, psychology, epistemology, management, pedagogy, and sociology to lay the foundation of Systems Medicine in Italy. This truly interdisciplinary group is attacking siloed approaches to medicine by integrating systems thinking in the three main aspects of real-world medicine: research, clinical care, and health care systems management.

Dr. Pristipino described the functioning of this most unlikely convergence through a well-defined structure of the organization that works through mixed working groups and task groups. Together, members are engaged in deep scientific advances such as the effect of quantum approaches, fuzzy inductive reasoning and network science to advance the delivery of Systems Medicine. The importance of clinical engagement with the patients as the center of care was also a key theme in Dr. Pristipino's talk, who emphasized the need for an all-around growth and collaborative mindset to ensure sustainability of medicine.

### Session 2: -omic technologies and complex diseases

#### Toward clinical decision support in oncology: Identifying driver alterations and therapeutic options (keynote address)

*Nikolaus Schultz, PhD,* from Memorial Sloan Kettering Cancer Center discussed oncological clinical decision support applications where clinical sequencing of tumors becomes a mainstream in cancer care. He discussed the various tools developed by his group for clinical support and how they helped identify the implications of specific mutations. Specific applications highlighted included the Cancer Hotspots database for single residue and in-frame indel mutation hotspots, OncoKB Precision Oncology Knowledge Base, and the cBioPortal for Cancer Genomics.^2–5^ It was stated that >42,000 of their institution's sequenced tumor samples were profiled by using these innovative and state-of-the-art tools, which represents a milestone in clinically relevant oncogenomics.

#### Comparative oncogenomics of liver disease: The sex matters

*Damjana Rozman, PhD*, from University of Ljubljana, Slovenia, presented how biological sex is an under-represented variable in liver pathologies. Her laboratory developed a mouse knockout with diminished cholesterol synthesis with a deleted *CYP51* gene. Novel substantial differences between female and male liver metabolism and gene expression patterns in HCC were discovered.

Specifically, female mice subgroups of HCC transcriptomes contained disbalanced cholesterol metabolism profiles, which were confirmed in human HCC transcriptome public databases. Disbalanced cholesterol metabolism was a risk factor for sex-dependent liver damage and more aggravating disease phenotypes in females. Identification of these trends opens venues toward personalized approaches in liver disease intervention strategies.

#### Metabolomic biomarkers predictive of radiation late effects

*Amrita Cheema, PhD,* of Georgetown University, Washington, DC, discussed the value of metabolomic markers to assess the late effects of radiation therapy in a cohort of prostate cancer patients that received treatment at Medstar Georgetown University Hospital. Findings from this initiative helped delineate robust biomarkers that predict radiation toxicity, including proctitis and tumor recurrence with high specificity and sensitivity. Radiation metabolomics was presented as a stand-alone technology to understand the molecular basis of perturbations due to radiation late effects.

### Panel discussion 1: Technology and regulation of digital medicine

Panel 1 was moderated by Robert Jarrin, JD, an adjunct assistant professor at Georgetown University School of Medicine. Panelists included Kapil Parakh, MD, adjunct assistant professor of medicine, Yale University, New Haven, CT and Medical Lead, Google Fit, Google, LLC; Sylvia Trujillo, MPP, JD, senior Washington Counsel, Life Sciences/Digital Medicine, The American Medical Association (AMA), Washington, DC; Pat Baird, MBA, head of Global Software Standards, Philips, Pleasant Prairie, WI; Bakul Patel, MBA, associate director for Digital Health, Center for Devices and Radiological Health, FDA, Silver Spring, MD.

The panel discussed current policy and regulatory developments in the exciting field of digital health and medicine. Topics included FDA regulation of digital medical devices, Medicare reimbursement for remote physiological monitoring treatment management services, Current Procedural Terminology (CPT^®^) coding of medical professional digital health services, and advances in technology, including AI and machine learning in Systems Medicine.

Panelists began by answering two simple questions: what is digital health and what is digital medicine? Mr. Patel spoke of FDA's institutional investments to bolster agency expertise in this evolving area, both internally across FDA and externally through industry and patient engagement. He addressed the nexus between medical device interoperability, privacy, security, and health information technologies and informatics. Ms. Trujillo gave an overview of AMA's roles in advancing the adoption of digital medical technologies while ensuring their safety, efficaciousness, and equity.

Dr. Parakh discussed the regulatory challenges that come from rapid evolution of technology as well as the importance of incorporating scientific evidence into digital health products. Mr. Baird provided an overview of the rigor behind technological standards specific to digital health and Systems Medicine. He argued that although standards are widely adopted, common challenges are not technological but rather rooted in design and usability flaws. The panel discussion was well received with several stimulating questions asked by the audience.

### Session 3: Ethics and educational considerations in the era of precision medicine

#### Ethics, education, and organization of health services in the era of big data (keynote address)

*Igor Svab, MD,* presented insights from the department of family medicine, University of Ljubljana, Slovenia. Dr. Svab outlined how big data poses considerable challenge to the profession of medicine and the health care system. There is a need for new educational directions, ethical considerations and structures for health care service delivery as greater numbers of personal genomes are integrated into various clinical specialties and health care service payment structures. New team compositions with greater participation of genetics counselors, bioinformaticians, and health data security experts must be achieved. Core educational content for clinicians in primary care and hospital settings across various medical specialties outlining minimum competence standards in the context of continuously evolving large-scale data complexities must be defined. Currently practicing providers must develop solutions for integrating new complex genomic diagnostic tools into routine clinical investigation. Integral to moving forward is the unified dialogue between specialist experts, medical providers, and allied health care professionals to use these advances to better patient care.

### Big data, brain science, and neuroethics: Expanding possibilities, addressing the problematic

*James Giordano, PhD,* from Georgetown University Medical Center addressed how big data approaches, combined with neuroscientific and neurotechnological advances, foster enhanced capacity to derive new insights to the structure and functions of the brain. Such capability affects definitions and meanings of normality and abnormality, health and disease, and the medical, social, economic, and legal regard, and treatment that these definitions evoke. In this light ethicolegal consideration of neuroscientific applications of big data must be carefully considered.

His team posits that effective ethical analysis and engagement of big data-dependent efforts in neuroscience require (1) assessment of actual capabilities and limits of neuroscientific and computational tools and techniques; (2) recognition of the ways that these methods, technologies, and outcomes can impact society—locally and internationally; and (3) a dialectic approach that is sensitive and responsive to cultural, philosophical, and ethical values and perspectives so as to develop a synthetic ethical framework for informing and guiding research, use(s)-in-practice, and relevant international policy.

### An ethical framework for the use of consumer generated data in health care

*Jessica Skopac, PhD, JD, MA,* a health policy analyst with the MITRE Corporation, outlined how consumer-generated data (CGD; e.g., social media use, Internet searches, buying behaviors, and memberships) are increasingly being used in predictive health care utilization analytics. The absence of ethical standards that clarify best practices presents implications for patient privacy and autonomy, trust-based patient–provider relationships, and could marginalize at risk individuals and populations.

Her team used a modified Delphi method to review U.S. and international CGD policies, health care ethics, ethical applications for analytics, algorithms, and machine learning to develop a final conceptual model, including five values (individual self-determination, health, distributive justice, trustworthiness, and privacy); eight principles (respect autonomy, consider fairness, ensure accountability, empower individuals and communities, preserve data security, promote data protection, promote transparency, and consider individual and population health); and 39 user-specific guidelines. For the framework to be flexible and broadly applicable to various situations, the values and principles are not articulated in any hierarchical order of importance, but rather require fact-finding and deliberation to determine whether the ethical permissibility of potential actions might involve weighting principles differently under different circumstances if they conflict.

An ethical framework is not an algorithm designed to recommend a certain course of action based on a set of variable inputs, but rather it is a tool to encourage reflection on important ethical concerns to improve conscientious decision making at every step of the process. This framework presents a lean but comprehensive structure that establishes a minimum ethical threshold for decision making about CGD use in health care—an ethical “safety net.” External expert review found this framework to be comprehensive, able to support user-specific ethical CGD decision making, and would assist organizations to safeguard persons and populations from negative impacts.

### Educational and ethical considerations for genetic test implementation within health care systems

*Emma Kurnat-Thoma, PhD, MS, RN,* from NIH, National Institute of Nursing Research (NINR) and Georgetown University School of Nursing and Health Studies, presented how the Precision Medicine/Precision Health (PM/PH) era presents unprecedented proliferation of genetic/genomic information and bioinformatic tools. Commercial sector trends for genetic test utilization in the U.S. health care system, health care provider workforce adequacy, genetics/genomics clinician education and training resources, and clinical decision support implementation resources were highlighted.

To ensure safe adoption and clinical translation of PM/PH, health care systems have an ethical responsibility to ensure their providers and frontline staff are adequately prepared to order, use, and interpret genetic test information. Strong partnerships between health care system leaders, frontline providers, and staff coupled with reasonable goal setting can help drive PM/PH translation interests.

Day 2 concluded with a stimulating poster session over wine and cheese. A total of 34 posters covering six themes were presented. A panel of judges picked three poster winners who were given award certificates at the concluding session of the conference.

## Day 3: Plenary Talks, Keynote Addresses, Panel Discussions, and Session Talks

### Plenary session 2, address 1: “Systems thinking and the U.S. population health ecosystem”

*William Rouse, PhD,* from the McCourt School of Public Policy at Georgetown University, gave the plenary talk on the concluding day. He discussed the fragmentation in the U.S. population health delivery systems and presented key challenges facing U.S. health care system, including substance abuse and the opioid epidemic, and the oncoming volume and complex health management needs of aging of baby boomers. He highlighted use of IT-enabled population health analytics strategies as a valuable tool to further understand these health system complexities and predict interventions to bring about their resolution.

The role of assistive technologies for disabled and older adults to enable information sharing and proper care coordination will facilitate higher value integrated health care. AI-based cognitive assistants will allow for deeper understanding of work domains and clinical workflows to better understand the preferences and needs of patients, disabled, and older adults. He concluded his plenary with a challenge and call for health care systems to use advanced analytic approaches to meet these needs.

### Plenary session 2, address 2: “Paradox entails new kinds of knowledge in medicine and elsewhere”

*Dr. Bruce West, PhD,* from the Army Research Office brought to light the paradox (a logical contradiction) that exists in the scientific modeling of complex phenomena in physical, social, or life sciences. Specifically, when paradoxes are encountered that a scientific community has not yet observed or encountered, how should conflicting experimental data and theoretical models be interpreted to understand the differences. He described ways to resolve this paradox and presented a new mathematical theory that addresses emergent properties by identifying macro variables for their description, which are independent of the dynamics of the micro variables they replace. Dr. West outlined how the collective behavior captured by the macro variables is often at variance with the more familiar reductionist theories with which we are more comfortable. He concludes that the emergent macro behavior resolves paradoxes and invariably produces a new way of thinking about familiar phenomena, one that could not be envisioned before the resolution. He showed how the self-organized temporal criticality model may formally overcome a paradox by replacing an either/or with a both/and way of thinking.

### Session 4: Drug repurposing and network medicine

#### Network medicine: The end of medicine as we know it? (keynote address)

*Harald H.H.W. Schmidt, MD, PhD, PharmD,* from Maastricht University presented the conceptual challenges and failures of the pharmaceutical industry since the 1950s in that most medications failed to provide a benefit to most patients, a logarithmic decline in efficacy and poor translation of basic biomedical research into medical applications. One reason for this was false academic incentives, that is, a focus on high-impact publications and funding income rather than patient benefit as ultimate measure of successful biomedical research. This has led to a lack of quality and reproducibility of published data, and a positive publication bias.

The biggest challenge that basic and clinical research needs to overcome is our current concept of a disease. We must shift our view of diseases from looking at them within one organ, to looking at them according to the underlying mechanism of action. He discussed through examples how Network Medicine will exploit to mechanism-based redefinitions and endophenotyping of diseases for network pharmacology, that is, synergistic combination therapy based on causal signaling modules. These modules are, however, distinct from current annotated pathways (e.g., KEGG and Wiki), which are not much more than mindmaps and not helpful in defining molecular disease mechanisms.

The key gap is not so much how many drugs will be able to be repurposed, but the development of precision diagnostics that allow for low intervention patient stratification. Examples of where this paradigm shift is being realized include redefinition of cancers, immune diseases, and a cluster of cerebro-cardio-metabolic phenotypes with clinical proof of concepts underway.

#### Pathway networks generated from human disease phenome

*Maricel G. Kann, PhD,* from University of Maryland Baltimore County presented a pathway-based approach to extend disease-variant associations and find new molecular connections between genetic mutations and diseases.

She used a compilation of >80,000 human genetic variants and their associated diseases to normalize phenotype terminologies in the Unified Medical Language System (UMLS). All variants were then grouped by UMLS Medical Subject Heading (MeSH) identifiers and enriched for Kyoto Encyclopedia of Genes and Genome (KEGG) pathways. Linking KEGG pathways to underlying genetic variations elucidated novel connections with disease phenome and metabolic pathways not detectable through gene-level analyses.

Examples included shared mutations between Noonan syndrome and essential hypertension, and common pathways in cardiovascular and connective tissue diseases. Identifying novel shared pathways across diseases constitutes an important contribution to extending disease-variant connections and provides the foundation to build novel disease–drug networks for new diagnostic and therapeutic interventions.

#### Network-based analysis to prioritize metabolic interventions in patients with anthracycline-induced cardiac dysfunction

*Feixiong Cheng, PhD,* from the Genomic Medicine Institute, Lerner Research Institute, at the Cleveland Clinic, introduced the concept of integrated network models and analysis to better understand comorbidity between diseases. Traditional epidemiology research designs are unable to fully account for the complexity of confounding genetic and environmental factors. Dr. Feng presented an innovative network methodology to understand the observed comorbidity with cancer treatments, specifically anthracycline-induced cardiac dysfunction (doxorubicin). A network-based methodology that focused on screening, monitoring, and treating anthracycline-related heart failure was developed.

He presented data from multiomic profiles within an integrated human protein–protein interactome, including transcriptomics from human-induced pluripotent stem cell-derived cardiomyocytes, metabolomics from doxorubicin-related cardiac dysfunction in rat models, and large-scale echocardiogram data from Cleveland Clinic's Epic database. Several novel pathways were identified, such as the AMP-activated protein kinase-signaling pathway. Highly integrative network analytic methodologies will allow for rapid identification and translation of metabolic interventions for anthracycline-induced cardiovascular complications.

### Session 5: Rare diseases and the role of systems/network medicine

#### The foundation for rare disease drug development in the era of systems medicine (keynote address)

*James Valentine, JD, MHS,* from Hyman, Phelps & McNamara PC, summarized the history and trends in approvals for therapeutic drugs by the U.S. FDA for the treatment of rare diseases for the past 35 years to present. Currently, 95% of all rare diseases are without an FDA-approved drug. Because 80% of rare diseases have a genetic origin, Mr. Valentine presented how Systems Medicine tools could be harnessed to bridge this gap through advancements in regulatory science.

He outlined how pioneering work at the U.S. FDA was being conducted to facilitate patient-focused drug development. Specifically, innovative regulatory reviews were conducted with the benefit of patient experience data—hearing directly from patients and caregivers as consultants, through patient-focused drug. Development meetings, and directly during qualitative video interviews during clinical trials—providing a systematic approach to ensure the needs of vulnerable patients suffering from rare diseases were met. A review of FDA-approval summary statistics for rare diseases identified effective and appropriate regulatory flexibility given the challenges and hurdles of this field.

#### Longitudinal profiling of carriers as a key to understanding human body homeostasis

*Ancha Baranova, PhD,* from George Mason University presented how traditional genetic analyses using knockout and knockdown animal models can limit progress in knowledge discovery due to time requirements and the inability to replicate findings in humans. High-throughput genome analysis allows for rapid scanning of individuals for the activation of a particular gene that can be correlated to a detectable phenotype.

Community-wide efforts in building database information containing precise genotype–phenotype correlations that are informed by quantified contributions of incomplete penetrance, haploinsufficiency, and heterozygous advantages are key to understanding human body homeostasis. Longitudinal profiling of parent carriers for children with autosomal recessive diseases that are greatly enriched in heterozygous variants represents a valuable stakeholder cohort for this effort.

#### Networks and tools for rare diseases systems medicine research

*Anne Pariser, MD,* from the NIH Office of Rare Disease Research (ORDR) at the National Center for Advancing Translational Sciences (NCATS) informed the attendees of programs, initiatives, and tools that are accelerating the development of rare disease treatments so that no patient is left behind. ORDR's programs included the Rare Diseases Clinical Research Network (RDCRN), which includes 20 centers of excellence, each of which focuses on three or more related rare disorders comprising a total of >200 different rare diseases.

The RDCRN funds innovative research to further multidisciplinary investigation of rare disease research, including natural history studies, clinical trials, and young investigator awards. The Data Management and Coordinating Center provides tools and common processes for the standardized collection of clinical research data for rare diseases. The Genetics and Rare Diseases Information Center provides clinical trial readiness grants, gene therapy platforms, in collaboration with the NCATS Therapeutics for Rare and Neglected Diseases program and microphysiological system “tissue chips” in the NCATS Division of Preclinical Innovation. They are also solving difficult translational hurdles and advancing the field of rare diseases research.

#### Patient stories and how they drive systems medicine research

*Christina Grant, MD, PhD,* from the Children's National Medical Center in Washington, DC, shared how the most intriguing patient stories can drive Systems Medicine research. Rare disease medicine faces the difficulty of small patient numbers in clinical trials, thus obscuring the cause and effective treatment of a disease.

Knowledge from patient stories coupled with data from biological systems and use of bioinformatics in rare disease research can help overcome limitations of small sample sizes and inform pathophysiology and therapies for more common conditions. Examples include repurposing a drug used in rare disease for a common disease based on shared biological networks; using a common medication to treat a rare disorder; using bioinformatics to more rapidly diagnose a rare disorder; and tailoring therapies based on combined knowledge from bioinformatics and biological networks.

#### Jeeva's AI-based virtual trials site accelerates clinical trials significantly reduces cost and patient travel burden

*Harsha Rajasimha, PhD,* described how his innovative startup company, JEEVA Informatics Solutions, Inc., used an AI-driven approach to overcome clinical trial operations obstacles stemming from travel burden when patients are not physically close enough to a research investigator to participate in eligibility, recruitment, and enrollment processes. Dr. Rajasimha outlined how using an AI-based virtual trial site helps investigators and directors of clinical trials operations accelerate speed, generate significant cost savings, and reduce participant travel burden by ∼80%. Geographically diverse patients, sponsors, and contract research organizations can include Jeeva as a virtual site in addition to brick and mortar sites as an option for patient participation in clinical trials through use of a smartphone-based technology platform, eConsent through database lock and study closeout, and eVisits. Jeeva's AI engine automatically learns from past clinical trials to guide and enhance overall success probability of future trials.

### Panel discussion 2: Current challenges in health care

The panel was moderated by Dinesh Verma, PhD, Stevens Institute of Technology. Panelists included William B. Rouse, PhD, Georgetown University, Washington, DC; Guru Madhavan, PhD, MBA, National Academy of Engineering, Washington, DC; Dennis McBride, PhD, SourceAmerica, Vienna, VA; Maeve McKean, JD/MSFS, Georgetown University Global Health Initiative, Washington, DC; and James C. Palmer, DMan, Caldwell Palmer, Denver, CO. The diversity of backgrounds of the panel members provided for a spirited discussion on domestic and global health and health care from a variety of points of view.

Professor Rouse stated that harnessing the use of Population Health principles, which can integrate health, education, and social services to keep a defined population healthy, will allow the United States to address health challenges holistically and better predict and support the realities of being mortal. The fragmentation of the U.S. health care delivery system is a formidable challenge, and Professor Rouse identified elements of the critical infrastructure that will be required to overcome this, such as strategic IT-enabled capabilities that foster health information sharing and patient care coordination. AI-based cognitive assistants that can provide advanced population analyses will allow for greater system-level prediction capabilities for at-risk subpopulations.

Dr. Madhavan remarked that metaphors and models from systems engineering can fruitfully aid in our understanding of the “state” of population health and medical systems. Using these approaches will allow us to understand both progress and degradations within the health care system, and to introduce proper accounting of expenditures and essential accountability for health outcomes.

Dr. Palmer stated that if he could, he would change the curriculum at all medical schools to include a focus on systems thinking and complexity science. He highlighted that although many speakers showed new ways to identify diseases (e.g., the notion of pathways), today, we lack a theory of humans as organisms. When we think of organizations, or extended organizations with distributed governance, they need to have the critical capability of self-awareness as a prerequisite to change.

Maeve McKean spoke to the closing gap between global health and domestic health. Drawing on the World Health Organization's Ten Threats to Global Health in 2019, Ms. McKean noted that for a majority of the burdens experienced by low-income countries were shared just as greatly by high-income ones. Examples included how both share the short- and long-term health effects of air pollution and climate change, changing demographics involving noncommunicable diseases, and the growing struggle with vaccine hesitancy. For each of these and others on the list, the “solution” requires shared partnerships and concerted attention from those within our own local and national health system, to engage in and develop structures for global information sharing and best practice implementation.

Dr. McBride questioned the rather universally accepted view that there actually is a medical or health care system—at least as systems scientists define systems. Properties of systems include regularity and order in the variables/relationships among inputs and outputs. In other words, systems are organizations of predictable feedback and feedforward systems. Many such systems adapt or learn. Dr. McBride believes that “health care” does not seem to align readily with these characteristics. However, if we use the taxonomy and methodology of *complex adaptive systems*, health care would be described as a *wicked system*.

A characteristic of such systems is that when attempts are made to “improve” system level performance, the treatment actually renders the system more complex, less predictable, and further degraded. Alternatively, we can use simplifying assumptions about key subsystems in the health care complex. This allows us to focus on problems such as diagnostic errors, which are cited as the third leading cause of death in the United States, and thus a legitimate intervention target. In this way, we can apply rigorous identification and examination of the patterns of subsystem interplay—and emergence—that in effect incubate diagnostic errors. Dr. McBride argued that there has been a paucity of examination of diagnostic error production at this level. There is an abundance of *organizational systems learning science* that can be applied toward improvements in diagnosis accuracy—that is, in the reduction of type I and type II errors, and the improvement of diagnostic timeliness.

### Session 6: AI/deep learning in medicine and natural language processing

#### Precisely practicing medicine from 700 trillion points of data (keynote address)

*Atul J. Butte, MD, PhD,* is the director of the Bakar Computational Health Sciences Institute, University of California San Francisco and chief data scientist for the entire University of California Health System (UC Health). Dr. Butte's laboratory builds and applies tools that convert “big data”—or trillions of points of molecular, clinical, and epidemiological data—measured by researchers and clinicians into diagnostics, therapeutics, and new insights into disease.

He presented how use of publicly available molecular measurements can be used to find new uses for drug therapies for autoimmune diseases and cancer; how big data can be mined for new “druggable” targets in disease; how to evaluate patients and populations presenting with whole-genome sequence data; integrating and reusing clinical and genomic data from clinical trials; discovery of new diagnostics for pregnancy complications; and most inspiringly, how the next biotech startup could come from your garage.

#### Learning to predict critical outcomes in the intensive care unit: The safe-intensive care unit perspective

*Tavpritesh Sethi, MBBS, PhD,* stated that a new machine learning article is published every 20 min, yet a small fraction of these articles impact patient care processes at the bedside. Dr. Sethi's research outlined his group's work to bridge this gap with Meaningful Enriching and Discovery-led AI for intensive care units (ICUs).

He presented case study data from predictive models for the ICU and public health settings, including the creation of Sepsis Advanced Forecasting Engine-ICU, the largest pediatric big data resource at All India Institute of Medical Sciences, New Delhi, India. The development of *wiseR*, an interpretable and interactive AI platform for constructing BDNs was highlighted, in addition to summaries of case studies for antimicrobial resistance and maternal health. Insights from these studies can inform future AI efforts in medicine to decrease U.S. health inequalities.

#### AI in radiological imaging: Lesson learned and possible roadmap ahead

*Seong K. Mun, PhD,* is a professor of physics from Virginia Tech, and director, Arlington Innovation Center for Health Research (Virginia Tech). During the past 30 years, radiology science developed computer-aided diagnosis using convolution neural networks (CNNs) before AI was a popular field. CNNs are a category of neural network algorithms that can take in images and assign weights and balances to various objects comprising the image to differentiate outputs.

Although the full potential of AI in imaging and informatics has yet to be realized, it is generating significant interest. Dr. Mun highlighted three primary obstacles to widespread use of AI in radiological practice: (1) current machine learning algorithms with CNN tools are based on handwriting and general images; however, radiology images require differentiation of subtle gray value features within local areas; (2) to build a usable CNN tool, a massive clinically representative data base with ground truths for extensive training and validation is required, but is extremely expensive to develop; and (3) the CNN tools in research settings do not translate easily into clinical settings and must take local clinical practice patterns and workflows into account.

## Conference Conclusion

Closing remarks and comments to the conference were provided by Dr. Harald H.H.W. Schmidt, MD, PhD, PharmD, who outlined key takeaways and highlights from the 3 days that spanned: Systems Medicine science, health care policy and health care system infrastructure, ethical legal and social considerations, health care provider education and training frameworks, population health analytics, clinical trial infrastructure, legal and regulatory considerations for drug development and technological innovations, big data, AI and machine learning.

Dr. Schmidt closed with a rousing invitation for attendees to attend the second international Systems Medicine conference held on March 18–20, 2020, in Munich Germany, and for presenters to consider submitting written work to the peer-reviewed journal *Systems Medicine.* The session concluded with final attendee comments, question/answer dialogue, and networking between speakers and attendees.

## Conference Website

https://sites.google.com/georgetown.edu/sysmedconf/home

## Notes

**Cite this article as:** Kurnat-Thoma E, Baranova A, Baird P, Brodsky E, Butte AJ, Cheema AK, Cheng F, Dutta S, Grant C, Giordano J, Maitland-van der Zee AH, Fridsma DB, Jarrin R, Kann MG, Keeney J, Loscalzo J, Madhavan G, Maron BA, McBride DK, McKean M, Mun SK, Palmer JC, Patel B, Parakh K, Pariser AR, Pristipino C, Radstake TRDJ, Rajasimha HK, Rouse WB, Rozman D, Saleh A, Schmidt HHHW, Schultz N, Sethi T, Silverman EK, Skopac J, Svab I, Trujillo S, Valentine JE, Verma D, West BJ, Vasudevan S (2020) Recent advances in systems and network medicine: meeting report from the first international conference in systems and network medicine, *Systems Medicine* 3:1, 22–35, DOI: 10.1089/sysm.2020.0001.

## Abbreviations Used

3D: three-dimensional
AMA: American Medical Association
ANF: antitumor necrosis factor
AI: artificial intelligence
BDN: Bayesian Decision Network
BCO: BioCompute Object
COPD: chronic obstructive pulmonary disease
CGD: consumer-generated data
CNN: convolution neural network
FDA: Food and Drug Administration
GWAS: Genome-Wide Association Study
HCC: hepatocellular carcinoma
ICU: intensive care unit
KEGG: Kyoto Encyclopedia of Genes and Genome
NCATS: National Center for Advancing Translational Sciences
ORDR: Office of Rare Disease Research
PM/PH: Precision Medicine/Precision Health
RDCRN: Rare Diseases Clinical Research Network
RCSB PDB: Research Collaboratory for Structural Bioinformatics Protein Data Bank
SNF: similarity network fusion
UMLS: Unified Medical Language System
